# Editorial to the Special Issue “Recent Advances in Biochemical Mechanisms of Acute Myeloid Leukemia”

**DOI:** 10.3390/biomedicines11051339

**Published:** 2023-05-02

**Authors:** Maria Mesuraca, Clelia Nisticò, Emanuela Chiarella

**Affiliations:** 1 Laboratory of Molecular Haematopoiesis and Stem Cell Biology, Department of Experimental and Clinical Medicine, University “Magna Græcia”, 88100 Catanzaro, Italy; mes@unicz.it; 2 Candiolo Cancer Institute, FPO–IRCCS and Department of Oncology, University of Torino, Strada Provinciale 142, km 3.95, Candiolo, 10060 Torino, Italy; nclelia2017@gmail.com

Acute myeloid leukemia (AML) is a clonal malignant disorder of myeloid progenitor cells characterized by uncontrolled proliferation, dysregulation in the differentiation program, and inhibition of apoptosis mechanisms. As a result, myeloid blasts cumulate in bone marrow, infiltrating many organs and tissues. Severe bone marrow impairment involves cytopenia at various levels with anemia, neutropenia and thrombocytopenia. Chronic fatigue, dyspnea from exertion, recurrent infections, malignant fever and bleeding characterize the course of the disease. For these reasons, AML progresses rapidly and is often associated to poor prognosis [1,2,3,4].

According to the latest data, the number of new AML cases per year amounts to 4.2 per 100,000 people [5].

In many cases, leukemia develops de novo, and the main risk factors are associated to advanced age, male sex, cigarette smoking and exposure to chemicals. In a limited number of patients, it occurs secondary to other hematopoietic disorders such as myelodysplastic syndrome or exposure to chemotherapy or high doses of radiotherapy [4,6,7].

According to the European Leukemia Net (ELN) and World Health Organization (WHO) guidelines, it is possible to stratify AML patients into three groups, favorable, intermediate and adverse, based on the cytogenetic profile of leukemia cells [8,9].

Usually, chromosome rearrangements involving t(8;21) (q22;q22.1), inv (16) (p13q22), or single mutations in *nucleophosmin 1* (*NPM1*) gene are associated to favorable risk, while alterations in *MLLT3 super elongation complex subunit* (*MLLT3*) genes deriving above all t(9;11) (p21.3;q23.3) as well as alterations in *fms-related receptor tyrosine kinase 3—internal tandem duplication* (*FLT3-ITD*) correlate with an intermediate risk [10,11]. On the other hand, deletion of chromosomes 5, 7 or 17, inversion of chromosome 3, translocation involving *BCR activator of RhoGEF and GTPase gene* and *ABL proto-oncogene 1* (*BCR-ABL*) t(9;22) (q34;q11.2) or mutations in several genes including *the tumor protein p53* (*TP53*), *runt-related transcription factor 1* (*RUNX1*), *additional sex combs like transcriptional regulator 1* (*ASXL1*), *BCL6 corepressor* (*BCOR*), *enhancer of zeste 2 polycomb repressive complex 2 subunit* (*EZH2*), *splicing factor 3b subunit 1* (*SF3B1*), *serine and arginine rich splicing factor 2* (*SRSF2*), *stromal antigen 2* (*STAG2*), *U2 small nuclear RNA auxiliary factor 1* (*U2AF1*), and/or *zinc finger CCCH-type, RNA-binding motif and serine/arginine rich 2* (*ZRSR2*) are involved in the cluster of adverse risk [10,12].

Today, scientific research is making great strides for the study of the molecular mechanisms that cause and support the aberrant proliferation of acute myeloid leukemia cells. For example, the consolidation of methodologies to simultaneously deliver or silence genes in primary health/tumor cells or cell lines offers the possibility to study alterations in glucose, lipid or nucleotide metabolism and then develop new therapies based on monoclonal antibodies or assist classic chemotherapeutics and/or radiotherapy with drugs targeting metabolic pathways (i.e., mevalonate signaling) [13,14,15,16,17,18].

Early diagnosis remains a nodal point to successfully treating AML or otherwise ensuring longer periods of remission.

The articles published in the Special Issue “Recent Advances in Biochemical Mechanisms of Acute Myeloid Leukemia” in *Biomedicines* journal offer new food for thought on the early diagnosis of AML pursued through innovative molecular tools that allow to investigate the immunophenotype of leukemia cells as well as variations in their transcriptomic profile compared to healthy cells.

In case of clinical suspicion of AML, the first exploratory approach is always the blood count and morphological analysis of peripheral blood cells and bone marrow cells. Today, the flow cytometric approach allows to rapidly investigate the phenotypic alterations in blood cells [19].

The immunophenotyping analysis in acute myeloid leukemia cells allows to identify the malignant expansion of blood cells according to their degree of maturation and lineage; specifically, the antigen patterns can be grouped into five clusters: precursor markers expressed on primitive hematopoietic stem cells (CD34, CD117, HLA-DR), myeloid markers typical of myeloid progenitor cells (cMPO, CD33, CD13), myeloid maturation markers associated to the terminal differentiation stages of granulocytes (CD11b, CD15, CD64, CD4, CD38, CD11c), monocytic markers (CD14, CD36, CD64), megakaryocytic markers (CD41, CD36, CD61), and red blood cells markers (CD235, CD71, CD36) [20].

However, immunophenotyping is not sufficient; an accurate diagnosis of AML aimed at personalized therapy requires an integrated use of multiple diagnostic tools including cytomorphology, cytochemistry, cytogenetics and molecular genetics techniques [21].

de Pinho Pessoa et al. analyzed a series of studies published in the last 10 years to clarify relationships between immunophenotyping and genetic changes in specific clusters of AML patients. In particular, the most frequent genetic abnormalities involving mutations on *NPM1*, *fms-related receptor tyrosine kinase (FLT3)*, and *DNA methyltransferase 3 alpha* (*DNMT3A*) genes are associated to dysregulation in CD34, major histocompatibility complex, class II, DR (HLA-DR), CD45, and CD13 cell surface antigen expression. Interestingly, lower levels of CD34 expression correlate with a favorable prognosis in NPM1-mutated AML patients since this is a typical marker of hematopoietic stem/progenitor cells. On the other hand, high levels of the pan-myeloid CD33 marker were detected in FLT3-ITD- mutated AML patients that unfortunately correlate with a shorter duration of remission. Regarding DNMT3A-mutated AML patients, the high proliferation rate of pre-leukemic cells is highlighted by higher levels of HLA- DR, Class II-associated invariant chain peptide (CLIP), the programmed cell death 1 ligand (PD-L1), and the T-cell immunoglobulin mucin family member 3 (TIM-3) antigens while CD34 marker is lower expressed. In this case, AML rapidly progresses due to dysregulation in oncogenes and/or tumor suppressor methylation [10].

In addition to the analysis of the immunophenotype, the study of the genome, transcriptome and proteome today are extremely relevant for diagnostic and, above all, prognostic purposes.

Ravi et al. analyzed the Transcriptomic Burden (TcB) to identify the biological progression of hematological malignancies and solid tumors in dogs. Their research strategy is based on the assumption that transcriptional complexity increases progressively with tumor progression, so transcriptomic burden also increases. Usually, in healthy cells, RNA levels are substantially higher than DNA levels, which is why cells need more time to synthesize them. When cells undergo neoplastic transformation, this constraint is removed and the RNA content increases gradually but progressively. The bioinformatic analysis on 21 different tumor types from 4179 canine cancer samples highlighted that the biological complexity of tumors decreased at increasing TcB levels, resulting in the presence of conserved biological patterns in both solid hematological malignancies. The TcBs analysis in a transcriptomic dataset of 657 pediatric solid extracranial tumors evidenced a significant increase in the global transcriptome, above all of cytokine activity and ECM genes, whereas TcB increased in normal tissue with a significant decrease in the genes involved in transcription regulation. In particular, a TcB shifting from low to high was demonstrated on pediatric Acute lymphoblastic leukemia (ALL) patients (out of the total 41 patients analyzed, 24 were characterized by a shift in TcB), indicating that the increase in TcB alone could be considered a hallmark of ALL progression. In addition, treatment of canine B cell lymphoma with phosphoinositide-3 kinase (PI3K) inhibitors, such as Buparlisib (BKM120), significantly decrease the TcB profile, thus configuring itself as a valid method to follow tumor progression and/or the efficiency of therapy [22].

To conclude, the integration of the classic methods for the diagnosis of AML with the most recent informative informatics methods could provide clinicians with a tool with very high diagnostic and prognostic value and aid in pursuing the goal of personalized anti-leukemic therapy, adequate for the transcriptomic profile of each patient.
